# Effect of the 3q26-coding oncogene *SEC62* as a potential prognostic marker in patients with ovarian neoplasia

**DOI:** 10.3389/fphys.2022.1054508

**Published:** 2023-01-04

**Authors:** Julia C. Radosa, Mariz Kasoha, Anne-Christine Schilz, Zoltan F. Takacs, Askin Kaya, Marc P. Radosa, Barbara Linxweiler, Maximilian Linxweiler, Rainer M. Bohle, Mathias Wagner, Gudrun Wagenpfeil, Erich-Franz Solomayer, Julia S. M. Zimmermann

**Affiliations:** ^1^ Department of Gynaecology, Obstetrics and Reproductive Medicine, Saarland University Hospital, Homburg, Saarland, Germany; ^2^ Department of Gynaecology and Obstetrics, Klinikum Bremen-Nord, Bremen, Germany; ^3^ Department of Otorhinolaryngologie and Head and Neck Surgery, Saarland University Hospital, Homburg, Germany; ^4^ Department of Pathology, Saarland University Hospital, Homburg, Germany; ^5^ Institute of Medical Biometry, Epidemiology and Medical Informatics, Saarland University Hospital, Homburg, Saarland, Germany

**Keywords:** *Sec62*, prognostic, therapy, ovarian cancer, borderline tumors of the ovary, tumor driver mutation, 3q26 amplification

## Abstract

With approximately 220,000 newly diagnosed cases per year, ovarian cancer is among the most frequently occurring cancers among women and the second leading cause of death from gynecological malignancies worldwide. About 70% of these cancers are diagnosed in advanced stages (FIGO IIB–IV), with a 5-year survival rate of 20–30%. Due to the poor prognosis of this disease, research has focused on its pathogenesis and the identification of prognostic factors. One possible approach for the identification of biological markers is the identification of tumor entity-specific genetic “driver mutations”. One such mutation is 3q26 amplification in the tumor driver *SEC62*, which has been identified as relevant to the pathogenesis of ovarian cancer. This study was conducted to investigate the role of *SEC62* in ovarian malignancies. Patients with ovarian neoplasias (borderline tumors of the ovary and ovarian cancer) who were treated between January 2007 and April 2019 at the Department of Gynecology and Obstetrics, Saarland University Hospital, were included in this retrospective study. *SEC62* expression in tumor tissue samples taken during clinical treatment was assessed immunohistochemically, with the calculation of immunoreactivity scores according to Remmele and Stegner, Pathologe, 1987, 8, 138–140. Correlations of *SEC62* expression with the TNM stage, histological subtype, tumor entity, and oncological outcomes (progression-free and overall survival) were examined. The sample comprised 167 patients (123 with ovarian cancer and 44 with borderline tumors of the ovary) with a median age of 60 (range, 15–87) years. At the time of diagnosis, 77 (46%) cases were FIGO stage III. All tissue slides showed *SEC62* overexpression in tumor cells and no *SEC62* expression in other cells. Median immunoreactivity scores were 8 (range, 2–12) for ovarian cancer and 9 (range, 4–12) for borderline tumors of the ovary. Patients with borderline tumors of the ovary as well as patients with ovarian cancer and an immunoreactive score (IRS) ≤ 9 showed an improved overall survival compared to those presenting with an IRS score >9 (*p* = 0.03). *SEC62* seems to be a prognostic biomarker for the overall survival of patients with ovarian malignancies.

## 1 Introduction

With approximately 220,000 newly diagnosed cases per year, ovarian cancer is among the most frequently occurring cancers in women; worldwide, it is the eighth leading cause of cancer-related death and the second leading cause of death from gynecological malignancies (Jayson et al., 2014; Siegel et al., 2016; Webb and jordan., 2017). Given the lack of early detection and screening options and their subtle presentation, 70–80% of ovarian carcinomas are diagnosed in advanced stages [International Federation of Gynecology and Obstetrics (FIGO) stages IIB–IV], with a 5-year survival rate of 20–30% (Buys et al., 2011; Torre et al., 2018). For this reason, and given the lack of tailored therapy despite the optimization of standard chemotherapeutic regimens, treatment options are limited. According to international guidelines, the standard treatment for ovarian cancer is maximal cytoreductive surgery followed by combination chemotherapy for many decades (Benedet et al., 2000). Ovarian cancer survival rates have hitherto been known to depend directly on the extent of surgical debulking, notably the achievement of complete cytoreduction (R0 resection), and on the amount of tumor remaining postoperatively (Bristow et al., 2002; Elattar et al., 2011). Due to the poor prognosis of this disease, recent research on ovarian cancer has focused on its pathogenesis, the identification of prognostic factors, and the development of precise therapeutic options based thereon.

A possible way of identifying these biologic markers is the identification of tumor-specific genetic tumor-driver mutations. Amplifications of the long arm of chromosome 3 (3q26 region) have been shown to be tumor-driver mutations, with high incidences in patients with head and neck, lung, and cervical cancers (Cancer Genome Atlas Research Network et al., 2008; Grobner et al., 2018). These alterations have also been observed with a frequency of 43.7% in patients with ovarian cancer (Hagerstrand et al., 2013). Given the potential impact of 3q amplifications on the pathogenesis of ovarian cancer, numerous studies have focused on the identification of 3q-oncogenes, such as *PIK3CA*, *p63*, *nCLAPM1*, and *FXR1*, but none of these genes has shown a functional correlation with ovarian tumorigenesis (Woenckhaus et al., 2002; Comtesse et al., 2007). Hagerstrand et al. conducted a systematic analysis of genes, which most frequently showed amplification in the 3q26 region and identified *SKIL* and *SEC62* as tumor-driver-genes for ovarian cancer development (Hagerstrand et al., 2013). *SEC62* encodes a transmembrane protein of the endoplasmic reticulum (Sec62). The precise physiological functions of the protein are not completely understood, but it has been shown to play roles in intracellular protein transport, ER-phagy to counteract cellular stress, and intracellular calcium homeostasis (Lakkaraju et al., 2012; Lang et al., 2012; Linxweiler et al., 2013; Fumagalli et al., 2016). *SEC62* overexpression in tumor tissue compared with tumor-free tissue has been observed for lung, prostate, and cervical cancers at the protein and mRNA levels, and high *SEC62* expression has been found to correlate with lymph node metastasis and poorer overall prognosis (Linxweiler et al., 2012, 2013, 2016; Wemmert et al., 2016). These results suggest that *SEC62* plays a role in the pathogenesis of these tumor entities and may be an important tumor-driver oncogene. Although details of the molecular mechanisms responsible for these functions are poorly understood, *in-vitro* experiments conducted with lung cancer cell lines have revealed increased stress tolerance and enhanced migration, transition, and proliferation of *SEC62*-overexpressing cells (Greiner et al., 2011).

In light of the findings of Hagerstrand et al. who identified *SEC62* as a potential tumor-driver-gene for the development of ovarian cancer and the prognostic impact of *SEC62* in other tumor entities (Hagerstrand et al., 2013), the aim of this study was to assess the role of *SEC62* as a possible prognostic marker in patients with ovarian neoplasia.

## 2 Materials and methods

### 2.1 Patients and tissue samples

All patients with primary ovarian cancer and borderline tumors of the ovary who were treated at the Department of Gynecology and Obstetrics, Saarland University Hospital, Homburg, Germany, between January 2007 and April 2019 were screened for enrollment in this retrospective study. The study protocol was approved by the Saarland Institutional Review Board (reference no. 207/11). The inclusion criteria were the availability of formalin-fixed, paraffin-embedded (FFPE) tissue samples and complete data on clinical parameters (including follow-up). Exclusion criteria were missing tissue samples and incomplete clinical information or follow-up. Data on patient and tumor characteristics were obtained by clinical chart review. Platinum sensitivity and resistance were defined as progression-free intervals of ≥6 and <6 months, respectively, after the completion of adjuvant platinum-based chemotherapy (Davis et al., 2014). *SEC62* expression was analyzed in the whole cohort and in patients with borderline ovarian tumors and ovarian cancer, respectively, and correlated with overall survival (OS) and progression-free survival (PFS).

### 2.2 Immunohistochemical analysis of *Sec62*

**FIGURE 1:**
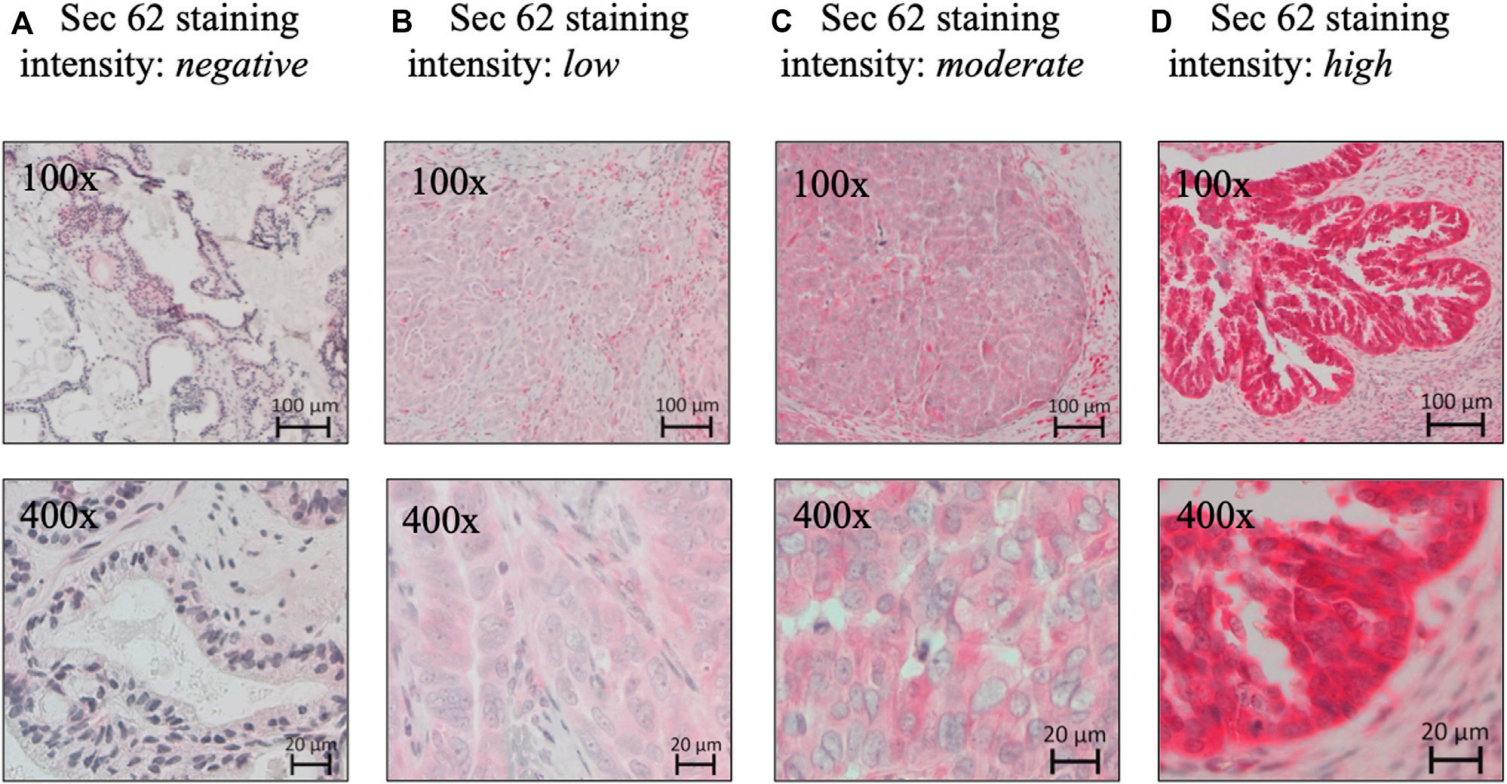
Sec62 immunohistochemistry. **(A)** Negative *SEC62* expression in normal ovarian tissue, as well as low **(B)**, moderate **(C)**, and high immunostaining intensity **(D)** in serous ovarian cancer. *SEC62* expression is indicated by a red signal, counterstaining with hematoxylin (blue).

**FIGURE 2:**
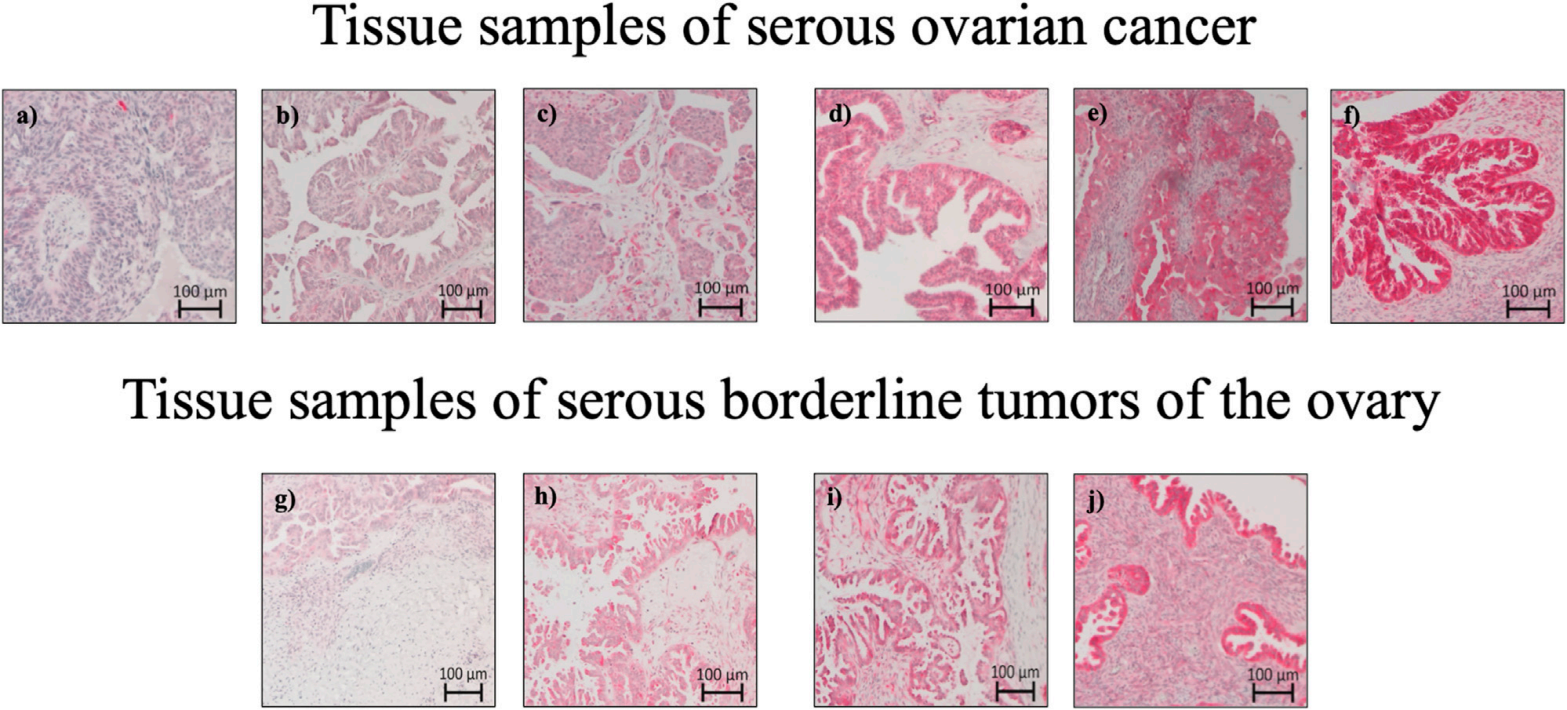
Sec62 immunohistochemistry stainings: Tissue samples of serous ovarian cancer and serous borderline tumors of the ovary. **(A)** Serous ovarian cancer with Sec62 immunoreactive score (IRS) 3, **(B)** serous ovarian cancer with Sec62 immunoreactive score (IRS) 4, **(C)** serous ovarian cancer with Sec62 immunoreactive score (IRS) 8, **(D,E)** serous ovarian cancer with Sec62 immunoreactive score (IRS) 9, **(F)** serous ovarian cancer with Sec62 immunoreactive score (IRS) 12, **(G)** serous borderline tumors of the ovary with Sec62 immunoreactive score (IRS) 4, **(H)** serous borderline tumors of the ovary with Sec62 immunoreactive score (IRS) 6, **(I)** serous borderline tumors of the ovary with Sec62 immunoreactive score (IRS) 8, **(J)** serous borderline tumors of the ovary with Sec62 immunoreactive score (IRS) 9.

A pathologist evaluated hematoxylin-stained tissue samples taken from representative FFPE blocks of the primary tumor specimens (definitive pathological specimens obtained during surgery) and histologically tumor-free ovarian tissue. The first three 10-µm sections of each sample were discarded, and 3-µm sections were then cut using a rotary microtome (RM 2235; Leica Microsystems, Wetzlar, Germany), transferred onto Superfrost Ultra Plus Microscope slides (Menzel-Gläser, Braunschweig, Germany), and dried overnight in an incubator at 37°C. After deparaffinization, heat-induced epitope retrieval was performed in retrieval solution (Dako S1699; Agilent Technologies, Santa Clara, CA, United States of America) and non-specific protein binding sites were blocked by incubation in a 3% bovine serum albumin (BSA)–phosphate buffered saline (PBS) solution (Sigma-Aldrich Chemie GmbH, Taufkirchen, Germany) for 30 min at room temperature. Subsequently, primary antibody incubation was performed with a 1:800 solution (diluted in 1% BSA–PBS) of a specific *SEC62* affinity-purified polyclonal rabbit antipeptide antibody directed against the C terminus of human *SEC62* (made in house) for 1 h at room temperature (Takacs et al., 2019). The rabbit antibody was directed against the COOH terminal undecapeptide of human Sec62 protein plus an aminoterminal cysteine (peptide sequence in single letter code: CGETPKSSHEKS). Commercial anti-Sec62 antibodies were described elsewhere as suitable alternatives (Liu et al., 2021). Each staining series included positive and negative (without primary antibody) controls. Visualization was performed using the Dako real detection system (Agilent Technologies) according to the manufacturer’s instructions, and the slides were counterstained with hematoxylin (Dako; Agilent Technologies, Glostrup, Denmark). Three independent examiners (one pathologist and two gynecologists) with wide experience in immunohistochemical (IHC) evaluation characterized *SEC62* immunoreactivity using Remmele and Stegner’s immunoreactive score (IRS), a well-established and unbiased semiquantitative validation system for the IHC assessment of estrogen receptor detection in breast cancer (Remmele and Stegner., 1987). In this system, staining intensity is classified as none (0), weak (1), intermediate (2), and strong (3). The percentage of stained cells is classified as none (0), <10% (1), 10–50% (2), 51–80% (3), and >80% (4). The IRS is the product of the staining intensity and stained cell percentage scores. We defined the samples’ Sec62 protein contents as low (IRS 0–8), and high (IRS 9–12), as in previous studies (Linxweiler et al., 2012; Wemmert et al., 2016) (Figure 1), and compared this content between tumor/borderline tumor tissue and histologically tumor-free tissue from the same patients (Figure 2).

### 2.3 Analysis of The Cancer Genome Atlas data

A dataset from The Cancer Genome Atlas (TCGA) was analyzed using publicly available sequencing data from the National Cancer Institute’s GDC data portal. The analysis was performed on 13 July 2022 and included 86,046 cases from 67 different primary tumor sites.

### 2.4 Statistical analysis

The SPSS software (v. 27; IBM Corporation, Armonk, NY, United States of America) was used for the statistical analysis. Qualitative and quantitative data are presented as absolute and relative frequencies and medians and ranges, respectively. For categorical variables (i.e., IRSs), we used Pearson’s chi-squared test for group comparison. The Kaplan–Meier method was used for the univariate analysis of PFS and OS durations (in months). Survival curves were compared using the log-rank test. Multivariate binary logistic regression analysis with stepwise forward and backward selection of factors associated with *SEC62* expression was conducted. Covariates for the multivariate analysis were selected based on the univariate findings and clinically relevant factors, such as the TNM and FIGO stages, histopathological subtype, platinum sensitivity or resistance, and tumor entity. Two-sided *p* values <0.05 were considered to be significant.

## 3 Results

To address the role of *SEC62* as a possible prognostic marker in patients with ovarian neoplasia, 171 patients with primary ovarian cancer and borderline tumors of the ovary, who were treated at the Department of Gynecology and Obstetrics, Saarland University Hospital, Homburg, Germany, were assessed for eligibility. Four of them were excluded due to insufficient slide quality and the lack of residual material for repeat staining. Thus, the analyses were conducted with samples from 167 patients (123 with invasive ovarian cancer and 44 with borderline tumors of the ovary). The median ages at the time of diagnosis were 62 (range, 15–86) years in the cohort of patients with invasive ovarian cancer and 53 (range, 21–78) years in the cohort of patients with borderline tumors (Table 1). At the time of diagnosis, 46% (*n* = 77) of patients had FIGO stage III G3 tumors and 77% (*n* = 129) of histopathological subtypes were serous (Table 2). The median PFS duration was 14 (range, 0–68) months and the median OS duration was 25 (range, 0–109) months (Table 3). Detailed oncologic outcomes are shown in Table 3.

**TABLE 1:** Patients’ characteristics (IRS = immunoreactive score) (*n* = 167).

	Invasive ovarian cancer (*n* = 123)	Borderline tumor (*n* = 44)	Whole cohort (*n* = 167)
Age [years (median; range)]	62 (15–86)	53 (21–87)	60 (15–87)
Menopausal status			
Premenopausal	18 (15%)	18 (41%)	37 (22%)
Postmenopausal	105 (85%)	26 (59%)	130 (78%)
FIGO stage			
I	23 (19%)	36 (82%)	59 (35%)
II	10 (8%)	5 (11%)	15 (9%)
III	74 (60%)	3 (7%)	77 (46%)
IV	16 (13%)	0 (0%)	16 (10%)
Chemotherapy			
Yes	92 (75%)	0 (0%)	92 (55%)
No	31 (25%)	44 (100%)	75 (45%)
BRCA germline mutation			
Yes	4 (3%)	0 (0%)	4 (2%)
No	2 (2%)	7 (16%)	9 (5%)
Unknown	117 (95%)	37 (84%)	154 (92%)
IRS score (median, range)	8 (2–12)	9 (4–12)	8 (2–12)

**TABLE 2:** Tumor characteristics (*n* = 167).

	Invasive ovarian cancer (*n* = 123)	Borderline tumor (*n* = 44)	Whole cohort (*n* = 167)
T stage			
1	27 (22%)	38 (86%)	65 (39%)
2	14 (11%)	3 (7%)	17 (10%)
3	82 (67%)	3 (7%)	85 (51%)
N stage			
0	76 (62%)	44 (100%)	120 (72%)
1	47 (38%)	0 (0%)	47 (28%)
R			
0	72 (59%)	44 (100%)	116 (69%)
1	35 (29%)	0 (0%)	35 (21%)
2	16 (13%)	0 (0%)	16 (10%)
Subtype			
Serous	99 (80%)	30 (68%)	129 (77%)
Mucinous	8 (7%)	14 (32%)	22 (13%)
Endometrioid	16 (13%)	0 (0%)	16 (10%)
Grading			
G1	5 (4%)	0 (0%)	5 (3%)
G2	41 (33%)	0 (0%)	41 (25%)
G3	77 (62%)	0 (0%)	77 (46%)
GB	0 (0%)	44 (100%)	44 (26%)

**TABLE 3:** Oncologic outcomes for patients with invasive ovarian cancer, borderline tumors of the ovary and the whole cohort (PFS = progression-free survival, OS = overall survival, *n* = 167).

	Invasive ovarian cancer (*n* = 123)	Borderline tumor (*n* = 44)	Whole cohort (*n* = 167)
Recurrence			
Yes	40 (33%)	3 (7%)	43 (26%)
No	83 (67%)	41 (93%)	124 (74%)
Distant metastasis			
Yes	43 (35%)	0 (0%)	43 (26%)
No	80 (65%)	44 (100%)	124 (74%)
Follow-up [months (median; range)]	28.5 (0–101)	51.5 (13–109)	35.5 (0–109)
PFS [months (median; range)]	14 (0–68)	17 (2–48)	14 (0–68)
OS [months (median; range)]	19 (–93)	51.5 (13–109)	25 (0–109)

### 3.1 Immunohistochemical (IHC) analysis of *Sec62*

**FIGURE 3:**
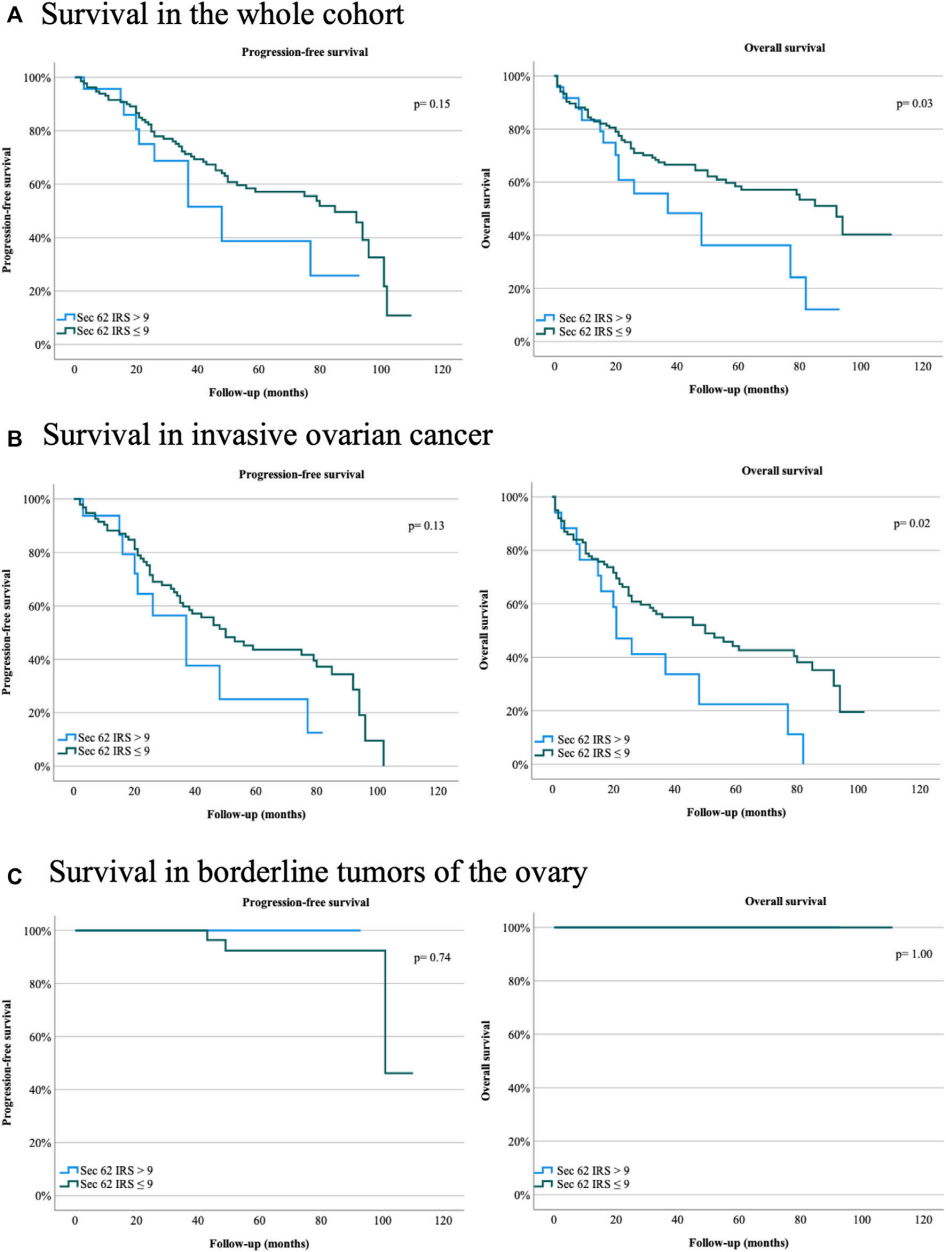
Survival rates for ovarian cancer patients and patients with borderline tumors of the ovary of the Department of Gynecology and Obstetrics, Saarland University Hospital, Homburg, Germany, between January 2007 and April 2019. **(A)** Progression-free and overall survival in the whole cohort. **(B)** Progression-free and overall survival in invasive ovarian cancer. **(C)** Progression-free and overall survival in borderline tumors of the ovary. Sec62 immunoreactive score (IRS) > 9, Sec62 IRS ≤9. Two-sided *p* values are indicated, values <0.05 were considered to be significant.

Immunohistochemical analysis was carried out according to an established protocol and employed an anti-Sec62 antibody, which is directed against the COOH terminal undecapeptide of human Sec62 protein and was already successfully used in various immunohistochemical analyses of human patient tissue (Greiner et al., 2011; Linxweiler et al., 2012; Bochen et al., 2017). Furthermore, the antibody had previously been shown to be specific for Sec62 under denaturing as well as native conditions, i.e. Western blot and fluorescence microscopy-signals were quenched after silencing of the *SEC62* gene in human cells (Greiner et al., 2011). All slides analyzed showed *SEC62* overexpression. We observed cytoplasmic Sec62 positivity in all ovarian cancer and borderline ovarian tumor cells, but not in physiological ovarian tissue cells. The median IRSs for ovarian cancer and borderline tumor samples [8 (range, 2–12) and 9 (range, 4–12), respectively] did not differ significantly (Table 1). No correlation between progression-free survival and Sec62 expression was detected when including the whole cohort, the cohort of patients with ovarian cancer or the cohort of patients with borderline tumors of the ovary respectively (whole cohort *p* = 0.15; ovarian cancer *p* = 0.13; borderline tumors of the ovary *p* = 0.74) (Table 4; Figure 3). Analyzing correlations between Sec62 expression scores and overall survival, we observed a median overall survival of 91 months (range 79–103) in the whole cohort consisting of patients with ovarian cancer and borderline tumor of the ovary with an IRS ≤9, while it amounted to 36 months (range 11–61) in patients with an IRS >9 (*p* = 0.03) Table 4, Figure 3A). For the cohort of patients with ovarian cancer, a median overall survival of 49 months (range 26–72) for the subgroup of IRS ≤9 vs. 20 months (range 12–28) for the subgroup of IRS >9 was observed (*p* = 0.02) Table 4, Figure 3B). No correlation between overall survival and Sec62 expression was detected in the borderline ovarian tumor cohort (*p* = 1.00, Table 4, Figure 3C).

**TABLE 4:** Oncologic outcomes in correlation with immunohistochemical SEC 62 expression (PFS = progression-free survival, OS = overall survival, IRS = immunoreactive score).

	Sec 62 IRS > 9	Sec 62 IRS ≤ 9	*p*
Whole cohort (*n* = 167)			
PFS (months, median, range)	47 (26–68)	84 (71–97)	0.15
OS (months, median, range)	36 (11–61)	91 (79–103)	0.03
Invasive ovarian cancer (*n* = 123)			
PFS (months, median, range)	36 (20–52)	49 (33–64)	0.13
OS (months, median, range)	20 (12–28)	49 (26–72)	0.02
Borderline tumor (*n* = 44)			
PFS (months, median, range)	44	48 (42–100)	0.74
OS (months, median, range)	92	109	1.00

### 3.2 TCGA data analysis

**FIGURE 4:**
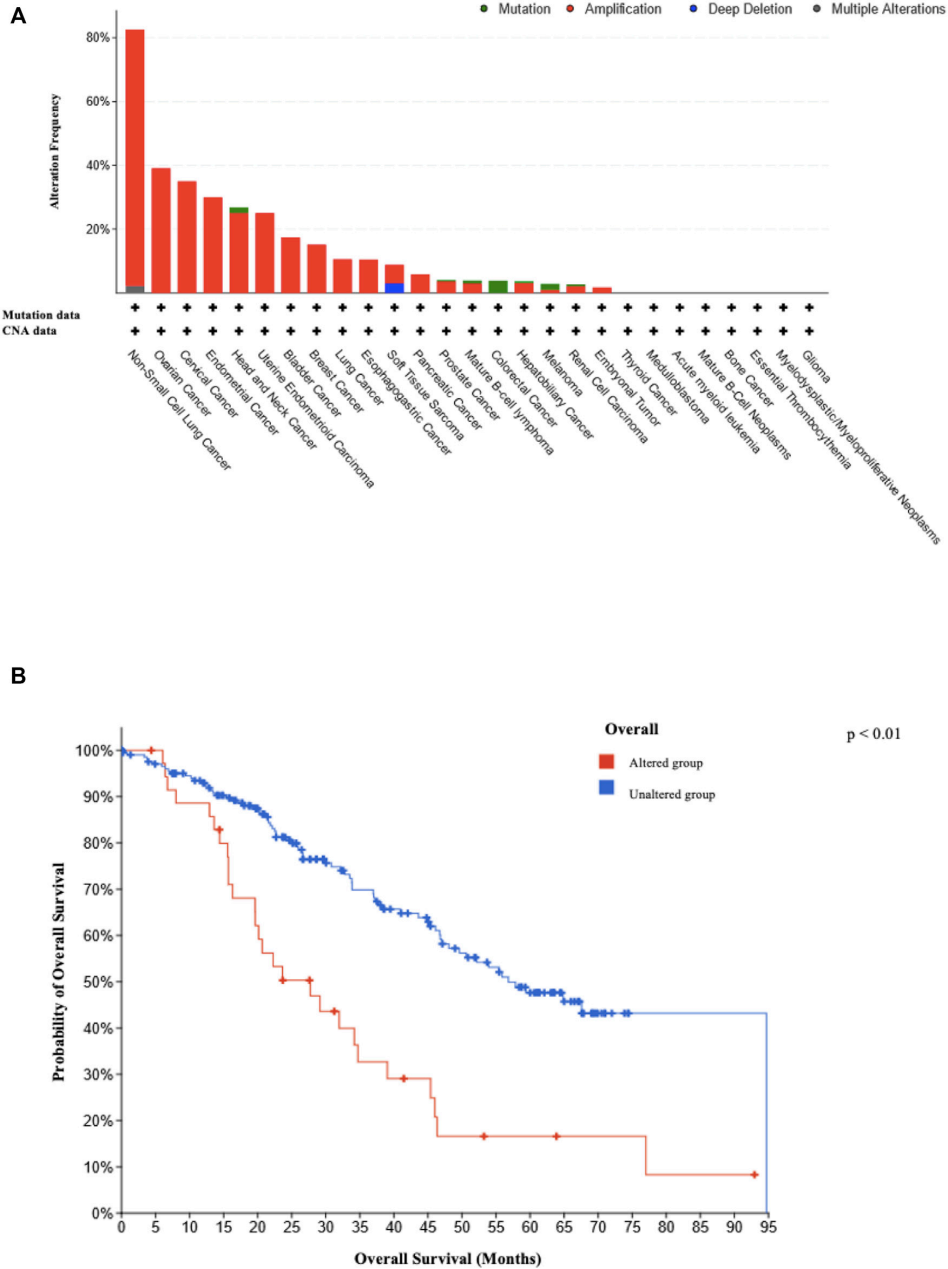
Type and frequency of reported *SEC62* gene alterations and related survival rates. **(A)** Type and frequency of *SEC62* gene alterations recorded in the TCGA atlas from the National Cancer Institute GDC Data Portal. The analysis was performed in 86,046 cases overall from 67 different primary tumor sites on 13 July 2022. CNA = copy number alteration **(B)** Overall survival across all cancer entities recorded in the TCGA atlas depending on *SEC62* alteration. The analysis was performed on 13 July 2022. Two-sided *p* values are indicated, values <0.05 were considered to be significant.

We observed *SEC62* alterations in 2,922 cases from TGCA, with a predominance of gene amplifications (Figure 4A). Thereby, five gynaecologic malignancies were ranked under the top eight tumor entities with the highest frequency of *SEC62* gene alterations (ovarian cancer (39% of cases), cervical cancer (35% of cases), endometrial cancer (30% of cases), uterine endometrioid carcinoma (25% of cases), and breast cancer (15% of cases)) (Figure 4A). Overall survival across all cancer entities recorded in the TCGA atlas was shorter in patients with *SEC62* alteration compared to patients without SEC62 alteration (*p* < 0.01; Figure 4B).

## 4 Discussion

In this study, we found that *SEC62* is overexpressed in ovarian cancer cells, *SEC62* expression can serve as a prognostic marker for patients with ovarian cancer or borderline tumor of the ovary and *SEC62* is a potential tumor-driver gene accounting for 3q amplification in ovarian cancer. For the prognostic role of *SEC62*, we could identify an IRS of >9 being associated with a shortened overall survival for patients with ovarian cancer, as well as for patients with borderline tumors of the ovary.

To our knowledge, this study is the first to address the prognostic relevance of *SEC62* and the correlation between *SEC62* overexpression and the poorer OS of patients with ovarian cancer. Our findings are in line with those for other tumor entities. Liu et al. observed a significant correlation of *SEC61G* overexpression with poor prognosis based on statistical analysis of data from the Cancer Genome Atlas cohort and the Chinese Glioma Genome Atlas cohort in patients with glioblastoma multiforme (Liu et al., 2019). Besides Bochen et al. found a high expression level of *SEC62*, defined as an IRS >0, to be significantly correlated with a shorter overall survival in IHC analyses of tissue specimens from 65 head and neck squamous cell carcinomas patients and 29 patients with cervical cancer of unknown primary (CUP) (Bochen et al., 2017). Overall survival was also shown to be significantly worse in a cohort of 53 patients with breast cancer, whose *SEC62* overexpression was assessed by Takacs et al. by also evaluating their Sec62 staining intensity by IHC analyses of tissue samples (Takacs et al., 2019). Takacs and colleagues defined an IRS cut-off of 8 for prognostic relevance, whereas we found a cut-off of 9 to represent overexpression and be prognostically relevant for OS (Takacs et al., 2019). This slight difference in cut-off scores seems to be of statistical interest and can be waived in terms of oncological outcomes, underlining the observation that IRS >9 seem to be associated with poorer overall survival in at least two gynecological cancer entities.

In contrast to others, we found no direct correlation in multivariate or Kaplan–Meier analyses between *SEC62* expression and the response to platinum-containing chemotherapy. In an analysis of 102 colorectal cancer tissue microarrays, Liu et al. found that *SEC62* promoted chemoresistance (Liu et al., 2021). *SEC62* seems to play a role in the response to chemotherapy, although only tendencies have been observed to date; further studies are needed to clarify this correlation.

Our analyses of the TCGA data, which showed alterations of this gene (mostly amplifications) in 39% of all cases, underlines the potential role of *SEC62* as a key tumor-driver gene in ovarian cancer. Hagerstrand et al. (2013) managed to expose the pathomechanism behind this tumor-driver gene, as these authors found *SEC62* overexpressing tumor cells to be characterized by increased proliferation and migratory as well as invasive potential, three hallmarks of cancer cells (Hanahan, 2022). The shortened overall survival across all tumor entities detected for the *SEC62* altered cohort emphasizes the prognostic effect of *SEC62*. These findings are well in line with other studies, in which *SEC62* was identified as an oncogene for lung cancer, prostate cancer and head and neck cancer and in which high *SEC62* expressions were correlated with a significant shorter disease-free and overall-survival (Greiner et al., 2011; Linxweiler et al., 2012 and 2016; Bochen et al., 2016).

With the identification of Sec62 as a potential prognostic marker for OS in patients with ovarian cancer, questions remain for further study. Due to the possible role of *SEC62* as a predictor of the response of ovarian cancer to platinum-based chemotherapy, prospective *in vitro* and *in vivo* studies are needed to move toward the implementation of this knowledge in daily clinical routine.

Limitations of this study are the lack of statistically evaluating interobserver variability, as interobserver variability is a known source of error in immunohistochemical studies. Besides, further studies using a second evidence tool, such as qPCR, should be implemented, to confirm immunoreactivity scores on the RNA level.

Future work with ovarian cancer cell lines will need to address the question of whether or not *SEC62* overexpression is associated with increased ER stress tolerance as well as increased migratory and invasive potential, three hallmarks of cancer that had been observed for various other *SEC62* overexpressing tumor cells (reviewed in this Research Topic by Zimmermann et al., 2022). Only if these *in vitro* experiments demonstrate a causative effect of *SEC62* overexpression on these three hallmarks in ovarian cancer cells, future *in vivo* experiments will address Sec62 as a potential therapeutic target for this tumor entity (also reviewed in this Research Topic by Zimmermann et al., 2022).

## 5 Conclusion

This study revealed an increased incidence of *SEC62* alterations and a correlation between high *SEC62* expression and worse OS in patients with ovarian cancer. These findings indicate that *SEC62* may play an oncogenic role in the pathogenesis of ovarian cancer.

## Data Availability

The raw data supporting the conclusion of this article will be made available by the authors, without undue reservation.
